# Continuous remote monitoring of neurophysiologic Immersion accurately predicts mood

**DOI:** 10.3389/fdgth.2024.1397557

**Published:** 2024-08-02

**Authors:** Sean H. Merritt, Paul J. Zak

**Affiliations:** ^1^ Center for Neuroeconomics Studies, Claremont Graduate University, Claremont, CA, United States; ^2^ Center for Neuroeconomics Studies and Drucker School of Management, Claremont Graduate University, Claremont, CA, United States

**Keywords:** mood, energy, mental health, emotional fitness, machine learning, prediction

## Abstract

Mental health professionals have relied primarily on clinical evaluations to identify *in vivo* pathology. As a result, mental health is largely reactive rather than proactive. In an effort to proactively assess mood, we collected continuous neurophysiologic data for ambulatory individuals 8–10 h a day at 1 Hz for 3 weeks (*N* = 24). Data were obtained using a commercial neuroscience platform (Immersion Neuroscience) that quantifies the neural value of social-emotional experiences. These data were related to self-reported mood and energy to assess their predictive accuracy. Statistical analyses quantified neurophysiologic troughs by the length and depth of social-emotional events with low values and neurophysiologic peaks as the complement. Participants in the study had an average of 2.25 (SD = 3.70, Min = 0, Max = 25) neurophysiologic troughs per day and 3.28 (SD = 3.97, Min = 0, Max = 25) peaks. The number of troughs and peaks predicted daily mood with 90% accuracy using least squares regressions and machine learning models. The analysis also showed that women were more prone to low mood compared to men. Our approach demonstrates that a simple count variable derived from a commercially-available platform is a viable way to assess low mood and low energy in populations vulnerable to mood disorders. In addition, peak Immersion events, which are mood-enhancing, may be an effective measure of thriving in adults.

## 1 Introduction

Depression and anxiety disorders are a growing health concern globally. For example, in the US, one in five people will face clinical depression in their lifetimes while one in 20 have had a depressive episode in the last year (1). Relatedly, 6.8 million U.S. adults annually experience an anxiety disorder while suicide rates have risen by about 30% since 2000 (2). Most recently, the COVID-19 pandemic was associated with substantial increases in depression (8.7% to 18.3%) and anxiety disorders [8.9%–22.6%; (3, 4)].

Mental health disorders are unequally distributed in the population. For example, anxiety and depression in adolescents increased by 10 percentage points from 2012 to 2018 (5, 6). Older populations do not fare much better with an estimated 20%–40% of the elderly experiencing depressive symptoms (7) and 17.1% suffering from anxiety (8). In the U.S. alone, the annual cost of depression exceeds $326 billion (9) while anxiety disorders drain between $20 and $45 billion from economic output (10).

Major depression occurs when one has low mood and a loss of interest in activities of daily living for at least two weeks (11). However, those with depression also have difficulty regulating their moods (12, 13). That is, people with clinical depression experience mood variability even while their average mood is low. Documenting mood variability is an important part of clinical practice because patients whose moods show less variability respond better to therapy (14). Similarly, those with anxiety disorders have been found to exhibit greater mood variability than individuals without this malady (15–17). Thus, mood variability may be a predictive indicator for the severity of depression and anxiety disorders. But, asking individuals to self-report moods hourly or more often is unlikely to produce useful data due to an inability to accurately report one's emotional states, the lack of an objective metric, and survey fatigue (18–20).

In addition to depression and anxiety, mood variability is associated with a number of detrimental behaviors including nicotine dependence (21, 22), alcohol abuse (23), and other substance abuse cravings (24). Those with mood variability are more likely to experience cognitive decline, including dementias, as they age (25). The incidence of suicidal ideation during a first or second psychotic episode increases with mood instability (26). Relatedly, swings in moods predict violent outbursts in psychiatric patients (27) and occurs in attention deficit hyperactivity disorder (28). Mood variability is also the prodrome for a variety of medical disorders including autoimmune diseases (29), heart failure (30, 31), Parkinson's Disease (32), Huntington's Disease (33), epilepsy (34), schizophrenia (35), and bulimia nervousa (36). Quantifying mood variability may thus predict multiple mental health and medical disorders (37).

The relationship between mood disorders and neural activity has been extensively researched (38–42). Irritable mood has been associated with increased amygdala activity (43) while sad mood has been related to increased activity in the ventrolateral prefrontal cortex (VLPFC), the anterior cingulate cortex (ACC) and related brain regions (44). Conversely, acute positive mood has been related to activity in the dorsolateral prefrontal cortex (DLPFC) and ACC (44). While it is important to know the neural sources of moods, these studies have limited clinical use as measurements are made using functional MRI for short periods in a laboratory setting.

There is substantial need for a predictive remote patient monitoring (RPM) solution for mood variations so that those at risk of mental health disorders can obtain help before they have a crisis. Yet, what should be measured and how often are unknown. Herein we report the results of a prospective observational study that sought to construct a predictive neurophysiologic measure for mood variations. Our goal is to provide clinicians with an objective, predictive measure indicating when individuals have pre-depressive symptoms, enabling early interventions. Catching disorders early nearly always improves treatment outcomes as well as reduces costs and patient distress (45). Yet, this has not been possible for mental health disorders because of the lack of diagnostic bioassays (46). We seek to empower patients and clinicians to better manage and treat mental health disorders by developing a predictive digital bioassay that is deployable at scale. In order to make this clinically useful, our analysis focused developing a simple metric that identifies when someone has the pre-depressive symptoms of low mood and low energy that can be tracked by patients and clinical teams.

We collected data from study participants at 1 Hz for 8–10 h a day, necessitating nontraditional analytical approaches as such data are typically random walks (47, 48). Machine learning (ML) is well-suited to such analytical challenges (49). ML models have been used to identify clinical depression using multiple behavioral and physiologic data with 70% to 80% accuracy (49, 50). Importantly, in prior research previous mood was a poor predictor of current mood indicating that self-reported data alone are unlikely to have sufficient predictive value (51).

Another challenge in predicting mood is the large number of possible variables that could be captured. For example, trait anxiety, diet, physical activity, stress, cognition, and sleep were found to be the most important features in a saturated model of depressed college students (52). Typically, in order to achieve high accuracy, ML models collect hundreds of measures. Most of these contribute little to predictive accuracy and increase the cost of data collection and processing. Moreover, such a broad-based approach makes it difficult to interpret the putative causal drivers of variations in mood states. Worse, the gold standard of predicting mood, variations out-of-sample, is rarely done in these analyses, adding uncertainty to the clinical value of such approaches (48).

A recent advance in predicting mood states used two neurophysiologic variables collected from a commercial neuroscience platform. These data predicted daily mood out-of-sample with 98% accuracy using ML (48). Parsimonious models using passive and continuous data collection provide clear indicators that identify pre-depression thresholds (48). Such linear indicators can trigger interventions that seek to alleviate persistent mood troughs.

The present contribution seeks to generate a clinically-useful indicator of mood variation using neurophysiologic data that we characterize by number and depth as well as by sex, age, time of day, and day of week. This level of detail is essential to understand who is at risk for low moods and when these are most likely to occur. To wit, women suffer a greater incidence of clinical depression compared to men (53–55) which is attributable to hormone variations, social factors, and in some cases the abuse women face (54, 55). A clinically-useful measure should be able to capture such factors by identifying differences in neurophysiologic responses. The approach we take here identifies neurophysiologic troughs and peaks statistically and then uses counts of these in order to predict of mood states. The use of a widely-available commercial neuroscience platform and a simple metric like counts of troughs and peaks is an effort to move our findings from research to clinical practice.

## 2 Methods

### 2.1 Procedure

Participants were recruited from a Texas residential living facility via flyers. Texas was as a convenient location with reasonably diverse population characteristics. A number of senior living residences in Texas were contacted for this study and the facility chosen was the one most open to recruitment of their residents for research. This project was approved by the Institutional Review Board of Claremont Graduate University (#1255) and follows the conditions in the Declaration of Helsinki. The research team arrived at the facility prior the commencement of the study and monitored data collection remotely thereafter. All participants who started the study completed it. Candidate participants provided written informed consent and were excluded if they had serious health issues; no exclusions were made.

Each participant was provided with an Apple Watch 6 with the Immersion Mobile app installed to collect neurophysiologic data during activities of daily living. Neurophysiologic data was collected over 20 days between January 18 and February 24, 2021 for 8–10 h per day. The data were averaged into daily observations (*N* = 480). Self-reported mood (“Mood”) and energy (“Energy”) were measured daily (see below), although some participants failed to make these reports, leaving a final sample of 404 observations obtained from 24 participants (72% female). The data collection burden for participants in this study was fairly high because they had to remember to charge their watches overnight every night, start the Immersion Mobile app every morning, and had to respond daily to surveys. As a result, only critical data were collected. The neurophysiologic data used in this study is the same as used in Merritt et al. (48) and the size effects from that paper result in a power of test for the present analysis of 0.99.

### 2.2 Self-reports

Two self-reported outcome measures were used in order to demonstrate robustness: Mood and Energy (capitalized to denote that these are variables). These data were collected each morning via email that requested the previous day's value in order to reduce the recency effect when reporting a daily average. Mood was measured by averaging four questions (cheerful, stressed, lonely, energy) on a 5 point Likert scale. Stressed and lonely were reverse coded using an abbreviated version of the Profile of Mood States [POMS (56, 57);] that has been extensively examined for its psychometrics properties (56). Averaging reduces variation, so as in previous research we isolated the variable Energy because it is a key indicator of depressive symptoms (58). Energy was measured with a single question. We defined a person having low Mood or Energy if their score was less than 3 (i.e., 1 or 2). A binary control variable measuring sickness (“Sick”) on the previous day was also collected.

### 2.3 Neurophysiology

Neurophysiologic responses were measured using a commercial platform (Immersion Neuroscience, Henderson, NV; https://www.getimmersion.com). Neurologic Immersion measures the value the brain obtains from social-emotional experiences by applying algorithms to data from the cranial nerves (48, 59, 60). Immersion has two main components, attention to the experience one is having, associated with dopamine binding to the prefrontal cortex, and the emotional resonance of the experience associated with oxytocin release from the brainstem (60–62).

The Immersion Neuroscience platform captures neuroelectrical activity associated with dopamine and oxytocin on the cranial nerves using a photoplethysmogram (PPG) sensor and algorithms applied to cardiac data (60, 63). These signals are convolved into a single measure called Immersion that was designed to accurately and consistently predict behaviors (59, 60, 64) The data were sent to the cloud continuously via participants' mobile phones and were also viewable in real-time. The Immersion Neuroscience platform provided an output file used in the analysis and no native data from participants' wearables were used in order to build parsimonious models.

We chose to measure neurologic Immersion for this study because of the well-established relationship between social interactions and mood (65). Moreover, by accessing neurophysiologic signals from smartwatches, continuous nonintrusive data were obtained from participants without affecting their daily activities (48, 60). In addition, the Immersion Neuroscience platform removes baseline physiologic responses each time it starts, automatically removes motion artifact, and interpolates missing data if collection is lost for less than one minute, thereby reducing the need for data cleaning.

**Figure A1:**
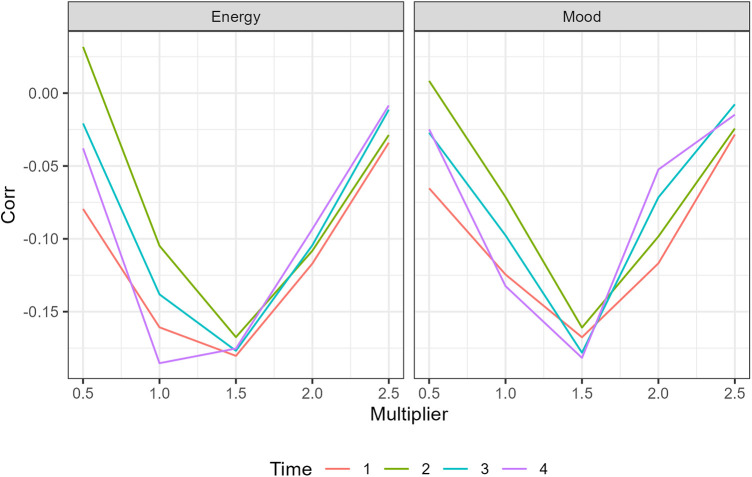
A graphic of the correlation with different trough definitions for a grid search of of a trough.

The first step in this analysis was to characterize Immersion troughs and peaks as previous research identified these as accurate predictors of mood (48). We focus on peaks and troughs because physiologic systems, including the nervous system, have a strong tendency to return to baseline. As a result, averaging Immersion over 8–10 h would not be expected to be predictive as the data would show strong mean reversion (66). There is little guidance in the published literature identifying when the value of a peripheral neurologic measure is considered atypical. As a result, we performed a grid search across the entire dataset to establish the length and depth of variations in Immersion that had the highest correlation with low Mood. This approach yielded a definition of an Immersion trough that will be used throughout the analysis: Trough = Immersion_it_ *<* *m_i_* *–* 1.5 ∗ *SD_i_* for at least 3 min, where Immersion_it_ indicates the Immersion of person *i* at time *t*, *m_i_* is the median Immersion of person *i*, and SD*_i_* is the standard deviation of Immersion for person *i*. For consistency, an Immersion peak was defined as Peak = Immersion_it_ *>* *m_i_* − 1*.*5 ∗ SD*_i_* for at least 3 min. The analytical details can be found in the Appendix (Figure A1).

### 2.4 Statistical approach

A variety of statistical methods were used to fully examine how the frequency and depth of peaks and troughs were related to Mood variations. The analyses began with t-tests, ANOVAs, and ordinary least squares (OLS) regressions and report measures of size effects (Cohen's d and *η*^2^). Participants with less than 4 h of data for a day were excluded. Daily variables included: the number of troughs, average trough time, average trough depth, average Immersion, number of peaks, average peak time, average peak height, whether person was sick, time between troughs, and time between peaks. The OLS models included daily fixed effects since troughs and peaks were constructed as differences from individual medians. Similarly we clustered the standard errors by day to avoid heteroskedasticity. OLS models were estimated in order to determine which candidate neurophysiologic variables to include in further analyses. The OLS estimations also examined the effects of sickness (Sick) and biological sex (Male) on Mood and Energy.

Next, ML models were estimated in order to quantify the predictive accuracy of the constructed neurophysiologic variables. Four different machine learning algorithms were tested: regularized logistic regression (logit), support vector machines (SVM), random forests (RF), and extreme gradient boosted trees (XGB). All machine learning models were done in Python with the *Sklearn* [version 1.1.2 (67);] and *XGBoost* [version 1.4.2 (68);] packages. A logit was included as it is the simplest model and serves as a baseline for fit and predictive accuracy. We also estimated SVM and RF models as they more effectively capture nonlinear responses as found in neurophysiologic data (69). XGB extends RF by training the parameter estimates on the residuals, generally improving accuracy (70). We assessed the most important features using a permutation technique in which a feature is randomly removed and the average decrement in accuracy is measured for 1,000 iterations (71).

The ML models were trained by transforming predictors into Z-scores. We then split the data into a training set (75%) and test set (25%). The current data were unbalanced for both low Mood (5%) and low Energy (21%). To address this, a synthetic minority oversampling technique was employed [SMOTE (72);] on the training set to avoid data leakage. We then tuned the hyper-parameters for each algorithm on the SMOTE training data using 5-fold cross validation (*GridSearchCV* function in the *Sklearn* package). The tuned hyperparameters can be found in the Appendix (Table A1).

The area under the receiver operator characteristic curve (AUC) was reported to compare ML models, as were accuracy (ACC), precision, and recall. AUC compares the true positive rate to the false positive rate and provides a balanced measure of model performance. ACC, the percentage of correctly identified observations, is the standard measure of model usefulness. The other two measures of model performance are precision, the true positive rate, and recall, the true negative rate. We report model performance for both the test set and the pre-SMOTE training data. A 5-fold cross validation (CV) was used to ensure that models were not over-fit using the entire SMOTE data. The 5-fold cross validation analysis measures the variation in performance of the model by splitting the data into 5 equal parts. The model is then trained with one split left out. The average score and standard deviation (SD) is reported for all models. In addition, to test if the model performed better than chance, a permutation test is used that randomly selects rows from 70% of the data (without replacement to avoid data leakage). A 5-fold cross-validation is then used to train the model which is used to predict the test data with the AUC recorded. We repeated this 100 times and performed a t-test to compare the distribution to random chance (AUC = .50).

The data are available at OpenICPSR-197830.

## 3 Results

The average daily number of troughs was 2.25 (SD = 3.70, Min = 0, Max = 25), while the average number of peaks was 3.28 (SD = 3.97, Min = 0, Max = 25). The number of troughs per day was positively correlated with the time spent in a trough (r = .63, *p* < .001) and negatively related to the depth of a trough (r = -.79, *p* < .001). Similarly, the number of peaks was correlated with peak height (r = .65, *p* < .001) and peak time (r = .72, *p* < .001). The number of peaks and troughs was negatively correlated showing consistency (r = -.27, *p* < .001).

We next tested if average trough depth and trough frequency varied by day of the week, time of day, and biological sex. We defined time of day as morning (7AM to 11 AM), afternoon (11 to 3PM), and evening (3 to 7PM). There were no differences for trough depth [F (6,211) = 1.14, *p* = .340, *η*^2^ = .003] or number of troughs [F (6,221) = 1.41, *p* = .213 *η*^2^ = .004] for different days of the week. The number of troughs varied by time of day [F (2,916) = 16.30, *p* < .001, *η*^2^ = .03] while average trough depth did not [F (2,916) = 2.36, *p* = .095, *η*^2^ = .005]. Participants had fewer troughs in the evening compared to other times of the day (*p* < .001; *p* < .001). There was also greater variation in number of troughs in the morning compared to the afternoon [F (361,368) = 0.593, *p* < .001] or evening [F (187,403) = 0.115, *p* < .001]. Evening and morning had similar variance in trough depth [F (187,368) = 0.856, *p* = 0.230] but troughs were larger in the afternoon [F (3,661,368) = 0.710, *p* = .001]. Finally, men and women did not differ in number of troughs [t (193.26) = −0.045, *p* = .964, d = 0.005] or depth of troughs [t (305.65) = −1.85, *p* = .066, d = −0.16], though women had more variation in number of troughs [F (280,87) = 1.79, *p* = .002] and average trough depth [F (280,87) = 4.28, *p* < .001] compared to men.

The daily characteristics of peaks were similar to troughs. The number of peaks [F (6,221) = 0.56, *p* = .762, *η*^2^ = .016] and average peak height [F (6,211) = 0.303, *p* = .935, *η*^2^ = .009] did not differ across days of the week, though the number of peaks varied throughout the day [F (2,916) = 10.45, *p* < .001, *η*^2^ = 0.02]. Morning and afternoon had the same number of peaks (*p* = .168) and both times had more peaks than evening (*p* < .001; *p* = .008). Average peak height did not vary by time of day [F (6,916) = 1.69, *p* = .186, *η*^2^ = .003]. The variation in number of peaks during morning was greater than the evenings [F (187,368) = 0.53, *p* < .001], but not the afternoon [F (361,368) = 1.11, *p* = .301]. Average peak height had higher variation in the morning than afternoon [F (361,368) = 1.69, *p* < .001] and evening [F (187,368) = 2.75, *p* < .001]. Men had a higher average peak height than women [t (365.31) = 4.42, *p* < .001, d = 0.33], but not more peaks [t (201.48) = 1.56, *p* = .120, d = 0.16], while women had greater variation in number of peaks [F (280,87) = 1.94, *p* < .001] and average peak height [F (280,87) = 12.15, *p* < .001].

### 3.1 Mood

Table 1 shows the average number of peaks and troughs for each Mood interval. As confirmed by the forthcoming statistical analysis, the Table shows the expected positive and increasing relationship between Mood and the number of Immersion peaks. Similarly, the number of Mood troughs is highest for low Mood and declines as Mood improves.

**Table 1:** Average peaks and troughs by mood level showing that higher moods are associated with a greater number of peaks and a fewer number of troughs.

Mood	Number of peaks	Number of troughs
1–1.9	0	6.00
2–2.9	2.29	2.43
3–3.9	2.61	2.82
4–4.9	3.78	1.93
5	3.76	1.20

There was a significant negative correlation between the number of troughs and Mood (r = -.19, *p* < .001). The reciprocal relationship was also significant with the number of Immersion peaks being positively associated with Mood (Mood: r = .20; *p* < .001).

To further examine these relationships and to identify which candidate neurophysiologic trough and peak variables to include in the machine learning models, six OLS models were estimated. Each neurophysiologic variable was evaluated separately to avoid multicollinearity and all six of the models control for sickness and sex. Consistent with our expectations, the number of troughs (b = −0.023, *p* = .037, CI = [−0.003,−0.04) and the number of peaks (b = 0.031, *p* = .003, CI = [0.013, 0.048) were related to Mood and carried the anticipated signs. Mood was also associated with the average time spent in troughs (b = −0.036, *p* = .015, CI = [−0.0097, −0.062) and peaks (b = 0.068, *p* = .022, CI = [0.015, 0.121) but was unrelated to average trough depth (b = −5.45, *p* = .264, CI = [−14.72, 3.82) or average peak height (b = 3.62, *p* = .259, CI = [−2.47, 9.71). In all six specifications, men were less likely to have low Mood than were women (Table 2).

**Table 2:** Ordinary least squares regressions relating neurophysiologic trough and peak variables to mood while including the control variables sick and male. The counts of troughs and peaks were significantly associated with Mood and carry the correct signs. Values in parentheses are standard errors.

	Dependent variable					
	Mood					
	(1)	(2)	(3)	(4)	(5)	(6)
Number of troughs	*−*0.023∗∗ (0.010)					
Number of peaks		0.031∗∗∗ (0.009)				
Avg. time in trough			*−*0.036∗∗ (0.014)			
Avg. time in peak				0.068∗∗ (0.027)		
Avg. trough depth					*−*5.448 (4.728)	
Avg. peak height						3.619 (3.106)
Sick	*−*1.024∗∗∗ (0.196)	*−*1.050∗∗∗ (0.186)	*−*1.049∗∗∗ (0.193)	*−*1.058∗∗∗ (0.188)	*−*1.047∗∗∗ (0.195)	*−*1.087∗∗∗ (0.192)
Male	0.202∗∗∗ (0.032)	0.222∗∗∗ (0.030)	0.210∗∗∗ (0.032)	0.200∗∗∗ (0.029)	0.195∗∗∗ (0.033)	0.219∗∗∗ (0.035)
Observations	369	369	369	369	369	369
R^2^	0.220	0.236	0.224	0.229	0.207	0.208
Adjusted R^2^	0.173	0.190	0.177	0.182	0.160	0.160

** *p* < .01.

*** *p* < .001.

### 3.2 Energy

The number of Immersion troughs had a negative correlation with Energy (r = -.19, *p* < .001) while the number of peak events increased Energy (r = .23, *p* < .001). Next, the same six OLS specifications as above were estimated using Energy as the dependent variable. As with Mood, number of troughs (b = −0.039, *p* = .004, CI = [−0.016, −0.062) and the number of peaks (b = 0.050, *p* < .001, CI = [−0.025, −0.075) were both statistically related to Energy levels and had the correct signs. Energy was also associated with average trough time (b = −0.054, *p* = .010, CI = −0.017, −0.092), average peak time (b = 0.105, *p* = .002, CI = [0.16, 0.049), and average trough depth (b = −13.83, *p* = .022, CI = [−24.69, −3.04). Only average peak time was unrelated to Energy (b = 7.572, *p* = .153, CI = [−2.38, 17.52). There was no sex difference for Energy levels (Table 3).

**Table 3:** Ordinary least squares regressions relating neurophysiologic trough and peak variables to energy, including the control variables sick and male. The number of troughs and peaks were significantly associated with energy and carry the correct signs. Values in parentheses are standard errors.

	Dependent variable					
	Energy					
	(1)	(2)	(3)	(4)	(5)	(6)
Number of troughs	*−*0.039∗∗∗ (0.012)					
Number of peaks		0.050∗∗∗ (0.013)				
Avg. time in trough			*−*0.054∗∗ (0.019)			
Avg. time in peak				0.105∗∗∗ (0.028)		
Avg. trough depth					*−*13.863∗∗ (5.524)	
Avg. peak height						7.572 (5.077)
Sick	*−*0.847∗∗∗ (0.174)	*−*0.890∗∗∗ (0.177)	*−*0.892∗∗∗ (0.163)	*−*0.905∗∗∗ (0.169)	*−*0.862∗∗∗ (0.165)	*−*0.957∗∗∗ (0.186)
Male	*−*0.0002 (0.062)	0.032 (0.063)	0.011 (0.063)	*−*0.004 (0.059)	*−*0.018 (0.063)	0.035 (0.061)
Observations	369	369	369	369	369	369
_R_2	0.126	0.146	0.127	0.133	0.119	0.116
Adjusted R^2^	0.073	0.095	0.074	0.081	0.065	0.062

** *p* < .01.

*** *p* < .001.

### 3.3 Machine learning

The significant variables in the OLS models were included in an ML estimation to assess their predictive accuracy using high or low Mood and Energy as dependent variables. Below we report the AUC for performance on the training set and observed (test) data. The full results can be found in Tables A2, A3 in the Appendix.

### 3.3.1 Mood

**Figure 1:**
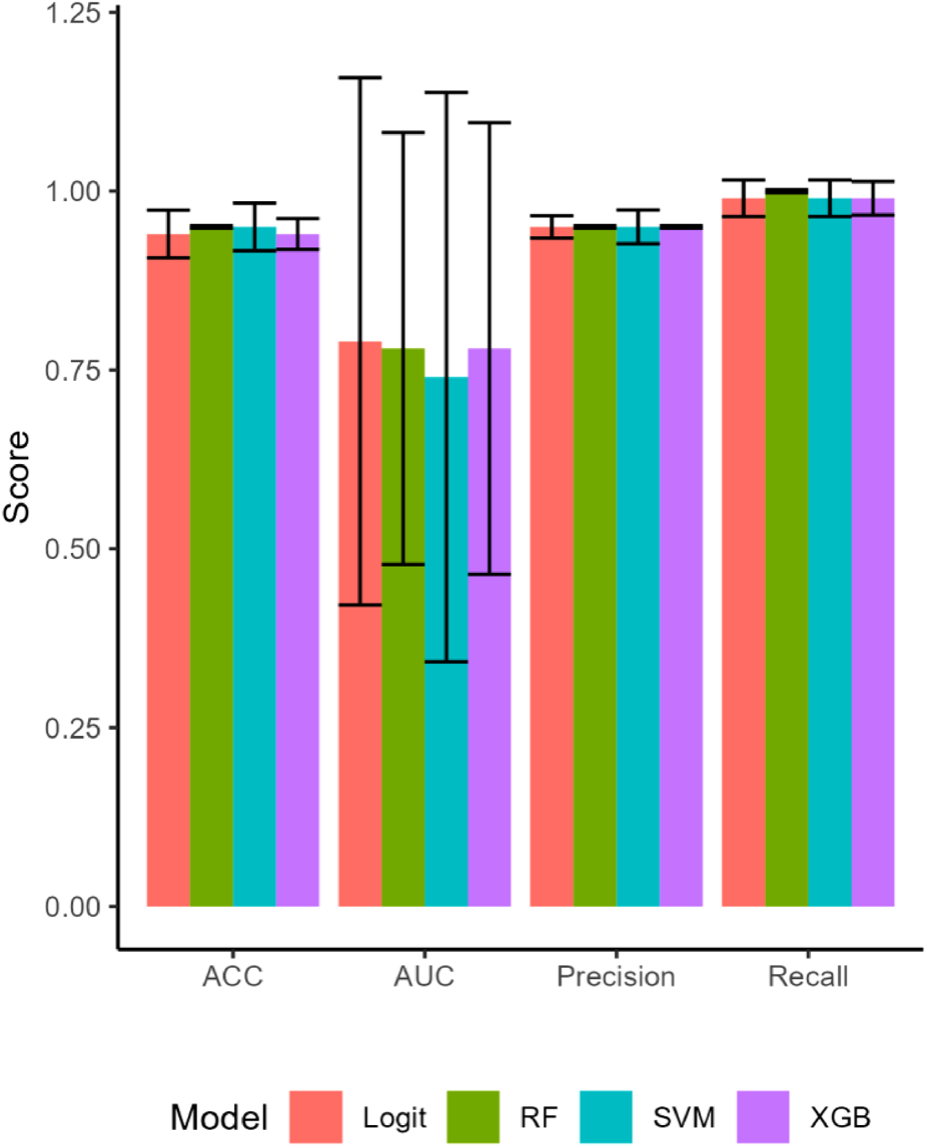
The attributes of the machine learning model estimations on the test set. All four models performed well for accuracy (ACC), fit the data well with high areas under the curve (AUC), and had high precision and recall. Bars are SEs.

The ML models predicted Mood accurately, with all four models having AUCs of 0.81 and higher for the training set. The predictive accuracy of the ML estimations on the test set for Mood fell for most models, but improved for the logit estimation (Figure 1). The logit model had an AUC of 0.95 and predicted Mood with 90% accuracy. This model also had perfect precision (1.00) and excellent recall (.90) (Table A2). Cross-validation confirmed that the logit model's performance was better than chance (t = 28.02, *p* < .001), as were the RF (t = 35.67, *p* < .001), and XGB (t = 26.99, *p* < .001) models.

### 3.3.2 Energy

The models classifying Energy using neurophysiologic variables performed well on the training set but under-performed the Mood models on the test set (AUCs 0.58–0.65; Table A3). Accuracy predicting Energy was similarly moderate at 67% for the logit model and 76% for the RF model. Cross-validation and permutation tests showed that the models' results were not due to chance (ps < .001).

## 4 Discussion

The analysis herein has demonstrated that continuous remote monitoring of mood using a commercial Neuroscience as a Service (NaaS) platform is feasible and highly accurate for this sample. Participants had their Immersion measured for 8–10 h a day at 1 Hz and had, on average, 2.25 troughs per day. As the number of troughs increased, the likelihood of experiencing low Mood increased linearly (r = -.19). This is confirmed by the linear relationship between the number of Immersion troughs and low Energy (r = -.19). Episodes of high Mood and high Energy were positively and linearly related to the number of peak Immersion events (Mood: r = .20; Energy: r = .23). Estimating a trained logistic regression model that included the number of peaks and troughs, sex, and a binary sickness indicator predicted Mood with 90% accuracy and was not overfit. Our SVM, RF, and XGB models performed well on the training model, but had large decreases in AUC and ACC in the test set. Given the results from the cross-validation, this is likely due to the randomized draw of the test set. The CV shows these models (with the exception of SVM) performed well on a held-out data set on average. Our findings for Energy confirmed the Mood results, but the predictive accuracy of Immersion peaks and troughs for Energy was lower.

We believe that having a continuous, passive, and accurate neurophysiologic indicator of low mood has significant clinical applications for psychiatrists and psychologists as well as primary care clinicians who are increasingly asked to evaluate patients' mental health (73) Indeed, our goal in developing a count variable for troughs was to make mood evaluations for clinicians rapid and unambiguous. In addition to accuracy, the use of an objective continuous measure of mood removes the recency bias inherent during in-person or telehealth visits (74).

Trough count data can be quickly reviewed for runs of days with, for example, the number of troughs at five or more indicating very low Mood and Energy. The clinician can inquire about these specific times to evaluate if pre-depression symptoms have arisen or if an outside factor has led to a temporarily low mood. For example, we showed that a clinician needs to inquire if the patient had been sick which can account for low Mood. We also showed that men are less likely to have low Mood compared to women consistent with findings for sex differences in depression (75). This initial analysis did not find differences by day of week, but did find more morning and afternoon peaks and fewer evening troughs. This shows the importance of all day data collection as truncated data may produce inaccurate indicators.

More generally, we posit that the number of peak Immersion events one has may be an adult measure of thriving. Immersion appears to be a neural measure of social-emotional value (48, 59, 60, 64). Social interactions increase Immersion and can induce peak Immersion events that we have shown improve Mood and Energy. By measuring the number of peak Immersion events, individuals and clinical teams may be able to assess if patients are thriving and, by offering ways to increase the neural value of social-emotional experiences, improve emotional fitness. Moreover, social withdrawal is a prodrome for a variety of disorders, including depression (12, 13), anxiety (15–17), schizophrenia (35), autoimmune diseases (29), heart failure (30, 31), Parkinson's Disease (32) and many others. At-risk patients could be invited to measure their Immersion and share the data with their clinical teams to assess when inventions are necessary; that is, when the number of peak events sufficiently declines indicating social withdrawal. In addition, the number of peak Immersion events could also be used to objectively evaluate whether clinical inventions improve patients' quality of life.

While the findings reported here are compelling and applicable, there are several limitations of the present study that require extensions. First, a larger and more diverse sample should be collected to confirm the predictors and thresholds we have reported. The Immersion Neuroscience platform removes baseline physiology from measurements, but this may be insufficient to control for variations in age, ethnicity, and personality traits. A larger sample will begin to resolve this issue. In addition, the length of data collection needs to be extended. Previous research using these data showed that Mood and Energy were predicted with high accuracy (≥92%) two days in advance of reporting (48). Immersion data collected for months or years could extend the accurate predictions of mood troughs to weeks or even months so that individuals who are vulnerable to mood disorders could share these data with friends or clinical teams to prompt check-ins. The ability of individuals to know, and have goals to build, their emotional fitness may be the most immediate extension of this work.

Additional research is also warranted before neurologic Immersion is ready for clinical use. For example, a baseline for Immersion troughs needs to be established for patients, starting with those suffering from mental health disorders, including those on selective serotonin reuptake inhibitors, serotonin and norepinephrine reuptake inhibitors, and related medications. Trough count thresholds for clinical depression are essential to transition psychiatry from reactive to proactive. Similar baselines and threshold should also be developed for those diagnosed with anxiety disorders, bipolar disorder, and medical syndromes associated with social withdrawal. Such data can lead to earlier medical interventions that are typically more efficacious than later treatments. Continuous digital bioassays, as we have shown here, are an effective way to reduce suffering and reduce medical expenditures.

## Data Availability

The datasets presented in this study can be found in online repositories. The names of the repository/repositories and accession number(s) can be found below: The data in this study can be found at OpenICPSR-197830.

## Appendix

The definition of a trough was established by identifying the relationship between Immersion and Mood. Individual *i*'s median Immersion (*m_i_*) was used to calculate immersion_it_ < *m_i_ −* λSD*_i_* on day *t* for at least X minutes. The values of parameter *λ* and time X were found using a 2-D grid search that identified the highest correlations with mood. The value of *λ* was varied from 0.1 to 2 in intervals of 0.1 and X was set to an integer as 1, 2, 3, or 4 min. The results from this grid search are shown in Figure A1. The grid search identified the optimal value for *λ* as 1.5. The Mood-trough relationship varied little with time, so 3 min was used as a reasonable mid-range value. This approach resulted in the definition of an Immersion trough as:

(1) $$\mathrm{immersio}n_{\mathrm{it}}<m_{i}-1.5*SD_{i}\;\mathrm{for}\;\mathrm{at}\;\mathrm{least}\;3\;\mathrm{minutes}$$

As shown in Figure A1 the same grid search yielded nearly identical values when examining correlations with Energy.

**Table A1:** Hyperparameters of the ML models.

ML	Parameter	Values
Logit	C	1, 10, 100
SVM	Regularization C	*l*_1_, *l*_2_, elasticnet 1, 10, 100
	Kernal	linear, polynomial, radial, sigmoid
RF	Min. sample slit	1, 2, 5, 10
	Min. sample per leaf	2, 5, 10, 15, 30
XGB	Max features number of estimators	sqrt, *log*_2_ 100, 500, 1,000
	Learning rate	0.05, 0.01
	Max depth	2, 4, 8
	Min child weight	1, 3, 5
	gamma	0.0, 0.15, 0.3
	Subsample	0.6, 0.75, 0.9
	sample of columns by tree	0.6, 0.75
	regularization of alpha	0.01, 0.1, 1

**Table A2:** The following covariates were used to predict mood: number of troughs, average trough time, average trough depth, average immersion, number of peaks, average peak time, average peak height, whether person was sick, time between troughs, and time between peaks. Values in parentheses are standard deviations from the cross validation. The figure shows permutation importance of the saturated model for Mood.

		AUC	ACC	Precision	Recall
Logit	Train	0.81	0.83	0.98	0.84
SVM		0.96	0.96	0.96	0.95
RF		1.00	1.00	1.00	0.99
XGB		1.00	0.99	1.00	0.99
Logit	Test	0.95	0.90	1.00	0.90
SVM		0.46	0.90	0.98	0.92
RF		0.47	0.92	0.98	0.95
XGB		0.71	0.90	0.99	0.91
Logit	CV	0.79	0.94	0.95	0.99
		(0.188)	(0.017)	(0.008)	(0.013)
SVM		0.74	0.95	0.95	0.99
		(0.203)	(0.017)	(0.012)	(0.013)
RF		0.78	0.95	0.95	1.00
		(0.154)	(0.0)	(0.0)	(0.0)
XGB		0.78	0.94	0.95	0.99
		(0.161)	(0.011)	(0.001)	(0.012)

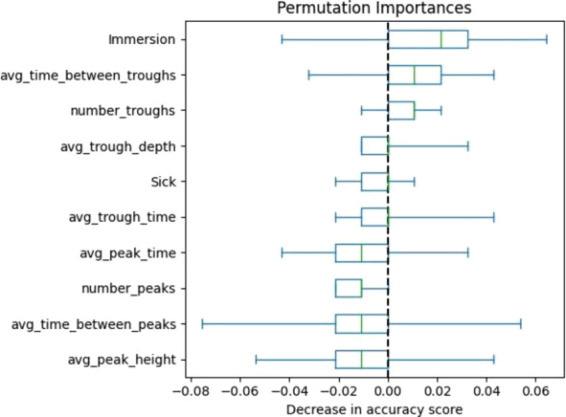

**Table A3:** The following covariates were used to predict energy: number of troughs, average trough time, average trough depth, average Immersion, number of peaks, average peak time, average peak height, whether person was sick, time between troughs, and time between peaks. Values in parentheses are standard deviations from the cross validation. The figure shows permutation importance of the saturated model for Energy.

		AUC	Accuracy	Precision	Recall
Logit	Train	0.69	0.71	0.89	0.72
SVM		0.80	0.80	0.83	0.75
RF		1.00	1.00	1.00	1.00
XGB		1.00	1.00	1.00	1.00
Logit	Test	0.65	0.67	0.88	0.68
SVM		0.57	0.66	0.83	0.72
RF		0.66	0.76	0.86	0.84
XGB		0.58	0.71	0.83	0.80
Logit	CV	0.67	0.80	0.80	0.98
		(0.1)	(0.03)	(0.021)	(0.023)
SVM		0.54	0.79	0.80	0.99
		(0.135)	(0.026)	(0.019)	(0.023)
RF		0.66	0.76	0.79	0.95
		(0.089)	(0.078)	(0.016)	(0.145)
XGB		0.69	0.76	0.79	0.95
		(0.105)	(0.072)	(0.013)	(0.125)
